# Biology Needs Philosophy, But What Philosophy?

**DOI:** 10.1093/biosci/biag016

**Published:** 2026-03-12

**Authors:** James DiFrisco, Steven Hecht Orzack

**Affiliations:** The Francis Crick Institute, London, UK; The Fresh Pond Research Institute, Cambridge, MA, USA

**Keywords:** philosophy of biology, theoretical biology, cell biology, evolutionary biology, philosophical biology

## Abstract

Philosophy of biology has the potential to contribute to biology by improving scientific reasoning. However, this potential is largely unrealized because of the lack of awareness by most biologists of what philosophy can offer, because of deficits of biological expertise among most philosophers, and because of adherence to disciplinary norms in philosophy that render much work in philosophy of biology irrelevant to biologists. We believe that philosophy of biology will contribute little to biology without a change of practice. We provide guidelines (“comments” *sensu* Slobodkin 1975, “commandments” *sensu* Kornberg 2000, 2003, Francis et al. 2007) for how biologists can better engage with philosophy and for how philosophers can better engage with biology so as to create a “philosophical biology” that improves our biological understanding.

Philosophy and science were once intertwined as “natural philosophy” (e.g., see Blair 2006, Grant 2007). Vestiges of this union can still be found in the journal titles *Philosophical Transactions of the Royal Society* and *Biological Reviews of the Cambridge Philosophical Society*. Although philosophy and science are now largely separate, we believe that something like “natural philosophy” can exist today and would improve the practice of science. Here, we focus on biology and philosophy of biology (POB) and how to create from them a “philosophical biology” that generates new and improved insights into nature.

In biology, there is increasing recognition of the need for guiding concepts and integrative theory (Phillips 2015, Nurse 2021, Levenstein et al. 2023, MacArthur 2023, Salguero-Gómez et al. 2024). The contribution of philosophers of biology to meeting this need could include connecting various kinds of knowledge, improving clarity of thought, and improving the formulation of biological hypotheses. Some have recently argued on these grounds that “science needs philosophy” (Laplane et al. 2019, p. 3948, Pradeu et al. 2023, Heger et al. 2024). However, the obstacles to philosophers of biology meeting the need for theory and concepts in biology are much greater than the advocates of integration suggest. Although many philosophers endorse “naturalism” (the view that science provides evidence and thus has authority over philosophy on topics of overlapping interest such as the nature of matter or evolution; see Papineau 2023), the idea that “science needs philosophy” appears to have little acceptance among biologists and other scientists.

We describe how attitudes and incentives within biology discourage engagement with conceptual and theoretical issues. We also describe why most POB is of little use to biologists due to deficits of biological knowledge on the part of the philosophers and because most work is tied to philosophical traditions that are irrelevant to biology. Philosophers of biology have tended to create a “duplicate space” of concepts that originate in biology but is too tenuously connected to biology to contribute to it. With the intention of improving the situation, we propose “comments” or metaphorical “commandments” (*sensu* Slobodkin 1975, Kornberg 2000, 2003, Francis et al. 2007, Elmer et al. 2017, Hulsen 2019, Turner 2020, Silva et al. 2021, Lyach 2022) for biologists and for philosophers who wish to contribute to philosophical biology. In doing so, we echo Francis and colleagues (p. 217) “….our humble intention is to stimulate much needed discussion.” Hereafter, we refer to these as “suggestions.”

## Why biology is not well connected to philosophy of biology

It is sensible to think that conceptual work in biology should be done by biologists. However, biology does not provide enough of this work to meet its needs (Woese 2004, Galas et al. 2008, Phillips 2015, Goldstein 2018, Nurse 2021, Levenstein et al. 2023, MacArthur 2023, Medzhitov 2026). We believe there are three reasons why this is the case.

The first is the attitude common among biologists that biology is primarily about the description and explication of facts about nature, such as how photosynthesis works. All biological investigations have a conceptual framework, but most often it is unacknowledged especially in print, as can be seen by perusal of an issue of, say, *Nature* or *Science*. This is especially true in cellular and molecular biology. Conceptual discussions in print are somewhat more common in ecology and evolutionary biology (e.g., Byrnes and Dee 2025, Nichols and Cooch 2025), but even there, conceptual discussions have often been disparaged by biologists who assert that the choice among different definitions of a concept is “arbitrary” or “semantic.” For example, Dobzhansky (1957, p. 235) writes of a controversy about the regulation of population size:

To a non-ecologist, the controversy which has made our session so lively is, I confess, somewhat bewildering. I have had a feeling for several years now that this is a controversy chiefly about words, about “semantics,” to use a fashionable word. Having tried to the best of my ability to understand the issue involved, I still continue to feel that way.

Dobzhansky later states that the controversy is not “futile” and that it stimulates “a lot of interesting research” but here he deprecates engagement with conceptual issues (see also Dobzhansky 1956). Of course, informal conversations among biologists about conceptual issues sometimes occur during lab meetings, coffee breaks, and post-work gatherings. Whatever their prevalence in the community of biologists, however, their informal venues underscore their secondary status, especially given the ever-increasing professional importance of publishing primary research articles. Publication of the description and explication of facts about nature without engagement with conceptual issues remains the central focus of almost all biologists.

The second reason for the public lack of engagement with conceptual issues is that many biologists view mathematical theories with suspicion (Wilson 1934, Thompson 1948, Mayr 1959, Slobodkin 1965, Bunnell 1973, Crick 1988, Kareiva 1989, Mitchison 2004, Roth 2011, Fawcett and Higginson 2012, Gibbons 2012, Kjeldsen 2017). This attitude is more common in cellular, developmental, and molecular biology despite the fact that mathematical theory has elucidated important phenomena such as receptors, ion channels, morphogens, and genes (see Gunawardena 2013). In some areas such as evolutionary biology and ecology, there is more acceptance of the need to refine concepts (such as natural selection) and of mathematical theorizing as being central to research.

The third reason is that advancement in many areas of biology is dependent on publication in journals such as *Cell, Nature*, and *Science*, which rarely publish conceptual articles. This reinforces the incentive to focus on the discovery and description of facts and the development of experimental methods. This typically diminishes engagement with the “big picture” of biology, although there are exceptions (see “Should philosophers of biology aim to contribute to philosophical biology?” below). Experimental work also typically requires staff and equipment. This creates an incentive to work on topics fundable by the National Science Foundation (NSF) and the National Institutes of Health in the United States and their equivalents elsewhere, which fund very few projects interfacing with POB. Instead, their mission is practical. For example, the mission of the NSF as described in their 2022–2026 Strategic Plan (2021, p. 10) is to “….make possible advances in everything from manufacturing and education to food production and health.”

These three attitudes can leave biologists unprepared to meaningfully address conceptual issues that *do* matter. An example of such an issue concerns sorting individual cells into types using single-cell RNA sequencing (scRNAseq) (Clevers et al. 2017). scRNAseq data provide an unbiased sample of all of the loci transcribed in individual cells. A standard practice is to apply dimensionality-reduction techniques so as to group cells into “clusters” based on their transcriptomic similarity. These clusters are taken to define cell types. This approach can be problematic. First, the membership of a cell in a cluster is sensitive to how dimension-reduction algorithms are parameterized, and current parameterization practices lack biological justification (Chari and Pachter 2023). Second, assignments to clusters can depend on batch effects, the sequencing methods used, and sequencing depth, even for the same cell sample (Domcke and Shendure 2023). This sensitivity means that clusters may not provide a stable classification system that can incorporate new data. Third, types based on transcriptomic similarity alone are not necessarily biologically interesting because their classification is not guided by relevance to a process of interest, such as physiological function, development, or evolution (Arendt et al. 2016, Domcke and Shendure 2023).

Progress on these issues will depend on clarification of how cell types should be defined theoretically, and on formulating dimensionality-reduction procedures to link the definition to its empirical measurement in scRNAseq data. This is a problem for biologists and philosophers to address. Biologists understand issues of definition to be of importance for making sense of transcriptomic data (Clevers et al. 2017). Philosophers can help answer questions of how measurements relate to theoretical concepts (cell types), and of how to ensure the meaningfulness of measurements (Chang 2004, Houle et al. 2011). They can also help answer questions of integration (Clarke 2010, Brigandt and Love 2012) such as “is it a problem when cell type classifications don’t overlap?” and “how can we know when to use which classification system?”

There are many topics that have benefitted or would benefit from contributions by biological theorists and philosophers of biology. Examples include how to extract causal information from black-box models of biological processes (Schölkopf 2022), assessing the role of hypotheses in data-driven research (Wang et al. 2023), understanding the relationship between gene regulation, self-organization, and mechanics in morphogenesis (Collinet and Lecuit 2021, Pfeifer et al. 2024, DiFrisco and Priya 2025), and understanding how novel body parts evolve (Brigandt and Love 2012, Erwin 2021).

Of course, it is not always the case that development and clarification of concepts are necessary. When a system is not well understood, ambiguity and plurality in concepts may be useful to biologists. For example, Rheinberger (2000, p. 222) notes, “The spectacular rise of molecular biology has come about without a comprehensive, exact, and rigid definition of what a gene is” (see also Crick 1988, Hull 1988, Robinson 2010, Griffiths and Stotz 2013, Pence 2024). Yet, a “fully” empiricist attitude neglects the essential role of concepts when generalizing over facts to find patterns. As Poincaré (1905, p. 101) writes: “Science is built up with facts, as a house is with stones; but a collection of facts is no more a science than a heap of stones is a house.” Facts are only organized into a coherent edifice through hypotheses and concepts.

In light of the above, we provide the following:

## Suggestions for biologists who want to contribute to philosophical biology

1. Understand that debate over definitions is often not quibbling over “mere” semantics. After all, semantics concerns meaning, and meaning connects concepts to inferential roles in reasoning, including prediction.
2. Understand that concepts having an uncertain connection with facts may still be useful. For example, the notion of a species as an ensemble of potentially interbreeding individuals has underwritten many important empirical insights into evolution (Mayr 1963), even though it can be hard to measure the potential for interbreeding over time and space. Similarly, the notion of an organ as a well-defined ensemble of cells has underwritten many important empirical insights in anatomy, pathology, and physiology even though the criteria that define organs remain in dispute (Minelli 2021).
3. Understand that there can be useful theory in biology even if it is not expressible in compact mathematical form.
4. Understand that theory can be important apart from its immediate empirical usefulness (Flexner 1939, Gunawardena 2013). However, theory that is informed by data and that informs data is most useful.
5. Understand that explanations of phenomena do not have to be molecular in order to be causal and mechanistic. The limits of explanations based upon molecular mechanisms do not necessitate switching to a different mode of explanation (e.g., one based on agency; see below).
6. Take guidance from philosophy when making *philosophical* claims. Debates by philosophers over issues such as falsifiability as a defining criterion of science; the uses of abduction, deduction, and induction; essentialism in classification; and the nature of scientific laws can improve scientific practice (e.g., see Raerinne 2024 and references therein).

We now describe an example of philosophical work by biologists that exemplifies mixed adherence to these suggestions. We do so in the spirit of Ladyman and Ross (2007), who described bad practice in philosophy in a way that focused on the content and not on the creators. Levins and Lewontin (1980, 1985) and Lewontin and Levins (2007) advocate for a “dialectical biology” where “dialectical” refers to a philosophical methodology derived from Hegel, Marx, and Engels. In their view (Lewontin and Levins 2007, p. 101), three dialectical principles should guide biological research: the “interchange of cause and effect,” “the negation of the negation,” and “the interpenetration of opposites.” Their work exemplifies adherence to suggestions 2, 3, 4, and 5. However, although they take some guidance from philosophy, they ignore the substantial literature in philosophy in which the dialectical understanding of reasoning and of nature itself is understood to be obscure (Russell 1947, Reichenbach 1951, Popper 1963, pp. 71–73, Elster 1985, Kitcher 1989, p. 265, Wright et al. 1992, p. 6). Perhaps this lack of adherence to suggestion 6 is justified because Levins and Lewontin believed the philosophical criticisms from Popper and others were invalid. If so, their silence fails the biologist and the philosopher interested in knowing their reasons for this belief. Some advocates of dialectical thinking were not silent in this way. For example, Cornforth (1968) addressed these criticisms by philosophers although not in the context of biology. The need to meaningfully engage with criticisms of dialectical biology is heightened by the conclusion of biologists Maynard Smith (1986), Simberloff (1987), and Shapiro (1993) that at best it has not produced biological insights that differ from those derived from other approaches (see also Lerner and Haldane 1938). Of these critiques, Lewontin and Levins (2007) only engage with Maynard Smith’s. In doing so, they note the equivalency of their three principles to familiar biological concepts: the “interchange of cause and effect” is “feedback” (p. 101), “the negation of the negation” is “non-linearity of change” (p. 108), and “the interpenetration of opposites” is exemplified by the interdependence of an organism and its environment (pp. 106–107). These equivalencies leave unresolved how dialectical biology leads to understanding of nature that is otherwise unattainable using extant, clearer approaches.

## Why philosophy of biology is not well connected to biology

POB is one of several disciplines (others are physics, chemistry, mathematics, and computer science) well suited to contribute to the clarification of ideas and to the formulation of hypotheses in biology (Orzack 2012, Pigliucci 2013, Pradeu et al. 2023). Yet such contributions are uncommon. Why is this? We suggest this can be understood in terms of norms and practices in the discipline of philosophy.

Philosophy addresses conceptual questions in many areas, including ethics, politics, and science. When a question is considered “philosophical,” the implication is that it cannot be resolved empirically. The question “how should causal claims be established and justified?” is answered differently from the question “does smoking cause lung cancer?” Training in philosophy does not involve mastery of empirical knowledge, but focuses instead on developing the capacity for conceptual reasoning and the formulation and evaluation of arguments. This capacity is primarily applied to problems that are historically considered important in philosophy, as reflected in Whitehead’s (1978, p. 39) comment that “The safest general characterization of the European philosophical tradition is that it consists of a series of footnotes to Plato.” Although an exaggeration, this captures the fact that the central problems of philosophy are set by traditions of the discipline more than by knowledge of empirical phenomena. The same is even true of POB. When considering the prospects for POB to contribute to philosophical biology, it is crucial to understand that most philosophical work is inward-looking in this manner. For example, there have been many articles and books in the philosophy of science in the last 100 years on the nature of “scientific explanation.” The reason is that certain early philosophers of science focused on this topic (Rosenberg 2005, Dewulf 2022) and *not* because it is the most important issue in science.

The reader may wonder how POB differs from “theoretical biology” as it is currently understood. One objective of POB is to use biological findings to question and revise the assumptions in debates within philosophy concerning, for example, the mind-body problem. A different objective is to investigate conceptual problems in biology so as to suggest changes of practice that would improve biological understanding. POB that pursues this latter objective of developing theory *for* biology overlaps with much of current theoretical biology. This second objective of POB should be part of the synthesis of biology and POB which we call philosophical biology.

How should philosophers of biology best contribute to philosophical biology? In Laplane and colleagues’ (2019) call for more philosophy in science, they argue (p. 3949) that this contribution includes “the clarification of scientific concepts” and “the formulation of new concepts and theories.”

## Clarification of scientific concepts

We agree that philosophical training could aid “the clarification of scientific concepts.” Advocates of this role for POB point out that philosophers of biology often focus on the concepts that biologists rely on, such as “mechanism,” “explanation,” “model,” and “individual,” which suggests that such philosophical work is relevant to biology (Heger et al. 2024). So why does it not influence biology more than it does? Merely addressing the same concepts is insufficient for making useful contributions. Understanding variations of a scientific concept and their inferential consequences requires comprehension of empirical facts and patterns (Sellars 1953, Brandom 2000, Wilson 2017). Such comprehension is rarely provided by philosophical training, and it is also not typically rewarded within philosophy (see below). As a result, much POB is characterized only by *apparent* thematic overlap with biology and is insufficiently grounded to provide useful clarification.

Although there is an opportunity for philosophers to contribute clarification, it is accessible only given knowledge of the relevant biology. What does this mean, given that *every* biologist lacks knowledge of substantial parts of biology? We suggest that the minimal knowledge required for philosophers of biology is that of a senior undergraduate major in biology. This level of knowledge means comprehension of central facts of biology and of their organization, which allows one to at least “know what you don’t know.” Such “metacognitive awareness” (Flavell 1979, Schraw and Dennison 1994, Kruger and Dunning 1999) is especially important in biology, given that the discipline has changed enormously in the last forty years. For example, evolutionary biology has been transformed conceptually by the study of molecular evolution. Yet a substantial portion of work in philosophy of evolutionary biology engages more with decades-old contributions (e.g., Lewontin 1970, 1983, Gould and Lewontin 1979) in part because they come from biologists who have supported POB as an endeavor. One consequence is that POB largely does not engage with many important conceptual issues in *current* evolutionary biology. For example, few philosophers engage with the modern understanding that selective and non-selective influences on a trait’s evolution need not be exclusive of one another. For example, Hartl and colleagues (1985) describe how natural selection leads to neutral molecular evolution. Finding no selective differences among alleles at a locus does not necessarily indicate that natural selection has not influenced the variation.

Many philosophers of biology appear not to have the level of comprehension of biology that we describe above. Many articles in POB contain incorrect claims about biology that are central to the argument. Even correct claims about biological facts are often presented as isolated examples, and without integration or synthesis. Lack of biological expertise among reviewers for philosophy journals likely contributes to this. One way is for philosophers and scientists to collaborate, although this may be difficult if they have divergent orientations and aims (see suggestion to philosophers 5 below). One way to address the problem is that POB submissions be reviewed by a biologist with relevant expertise as well as by a philosopher (see suggestion to philosophers 6 below).

Beyond the lack of adequate biological comprehension, another reason why philosophers have produced little useful clarification of biological concepts has to do with the inward orientation of philosophy. Hull (1969, p. 163) writes, “Philosophers have not been motivated in their choice of topics by any concern with issues currently of interest to biologists.” Fortunately, this is much less true now. However, POB is still mostly directed toward an audience of other philosophers rather than toward biologists. For example, there has been much work in POB analyzing the nature of “mechanistic explanation” (e.g., Bechtel and Richardson 1992, Machamer et al. 2000, Glennan 2017). This project aims to describe practices in biology in order to develop a theory of mechanistic explanation for philosophy of science. As such, it tends not to yield guidance or theory that can usefully influence biological research, although many biologists do regard understanding mechanistic explanations as important (e.g., Ozpolat et al. 2025). Even contributions by philosophers to this literature that are exemplary in their engagement with biology (e.g., Bechtel 2006) appear to be directed toward philosophers and are little known to biologists (see below).

The inward orientation of POB sets up a dynamic in which facts and concepts enter from biology and are transformed into objects of philosophical discussion that form a self-referential debate within POB. This process of “domestication” (Ladyman and Ross 2007) can be observed in reports of specific empirical findings as well as in entire topical areas of the philosophical literature. Biological domestication is the modification of animals and plants to serve the purposes of the domesticator. Likewise, domestication in the present context is the modification of facts or concepts so as to serve one discipline without necessary regard to the facts or concepts as understood by the source discipline. An example of domestication in philosophy concerns egg-laying behavior of species of wasps in genera such as *Sphex* and *Ammophila*. Wooldridge (1963) described this behavior as being “mechanical” in the sense of being repetitive and “unintelligent.” He based this solely on Fabre (1879), despite there being longstanding contradictory findings in the literature (see Keijzer 2013). Yet, the mechanical description then took on a life of its own in philosophy and cognitive science, being invoked repeatedly as fact by Dennett (1978, 1984, 1998), Hofstadter (1985), and others.

Examples of topical domestication include biological functions, mechanisms, fitness, and the “Extended Evolutionary Synthesis.” For example, a substantial amount of work in POB has focused on analyzing biological functions and purpose. Most of this work starts from the Darwinian principle that natural selection is the basis for ascribing function to traits. Evolutionary biology has long had quantitative theories of selection and adaptation (Walsh and Lynch 2018) that improve on the qualitative concept of function. Yet, the philosophical literature on biological functions has followed a separate track of qualitative conceptual analysis in which results from evolutionary biology play virtually no role. Biological functions are treated as a *philosophical* issue, and biology only enters as a source of examples or counterexamples for proposed definitions of function (e.g., Millikan 1987, Neander 1991, Mossio et al. 2009, Garson 2019). These exercises have not been influential in biology. We think they could have been if they had been carried out with deeper engagement with biology and with biologists.

## Formulation of new concepts and theories

We also agree with Laplane and colleagues (2019) that philosophical training could aid “the formulation of new concepts and theories” and with their implication that this has largely yet to occur. However, they do not mention that an important component in the development of many biological concepts and theories is the elaboration of how to connect them to data so that they can be confirmed or disconfirmed (Winsberg 1999). Many topics ripe for philosophical contributions, such as cell types, require an engagement with data and a level of numeracy that usually does not come from philosophical training.

Of course, some philosophical work has influenced biology. One example is Woodger (1937) in which symbolic logic is used “…. in order to construct a metalanguage for biology that would help organize biological statements and uncover connections between different biological theories” (Nicholson and Gawne 2014, p. 272). This work along with Gregg’s (1954) further application of Woodger’s ideas to taxonomy influenced the founder of cladistics Willi Hennig (1966, p. 16) who described their work as “extraordinarily important” for understanding hierarchical classification (Varma 2016). Another example is the work of Woodger and Joseph and Dorothy Needham, Dorothy Wrinch, John Bernal, and Conrad Waddington in the 1930s to build an experimental approach based upon Whitehead’s claim that (Peterson 2016, p. 12) “…the universe was not constructed primarily of isolated particles or mechanisms but relational wholes or *organisms*” (see also Abir-Am 1987).

Perhaps the most successful earlier contribution from philosophy to biology is McCulloch and Pitts (1943), whose model of the nervous system is the conceptual basis for what are now called neural networks. Their model is based on mathematical logic, and they cite only work by Carnap, Hilbert and Ackermann, and Russell and Whitehead (see Abraham 2002 for details). The influence of their article can be seen by examining citations of it, as found in the Web of Science, which compiles citations of articles, books, and theses found in thousands of journals and theses in science, the social sciences, and the humanities. Interpreting citation counts as an indication of conceptual influence can be problematic (Bornmann and Daniel 2008), but at minimum, they indicate what authors are aware of. As shown in table 1, there are 3858 citations, of which 585 are in journals or theses (“venues”) whose primary readership is biologists (e.g., *Cell*) or in a general science journal such as PNAS whose readership includes many biologists, 48 are in venues whose readership is composed of philosophers (e.g., *Philosophy of Science*), and 3225 in venues in non-biological scientific disciplines (e.g., *Computer Optics*). It is extraordinary how many more citations are found in biological venues than in philosophy venues. Of course, citations in non-biological scientific venues reflect awareness by other kinds of scientists of McCulloch and Pitts’s article.

**Table 1.** Citations of POB articles or books as recorded in the Web of Science, as of 9 September 2025.

Article	Biology	Philosophy	Other
McCulloch and Pitts (1943)	585	48	3225
Hull (1976)	260	191	30
Sober (1984)	291	469	113
Sober (1988)	262	190	52
Dennett (1995)	39	103	156
Matthen and Ariew (2002)	27	150	10
Bechtel (2006)	32	146	63
Okasha (2006)	507	251	115
Godfrey-Smith (2009)	219	357	52
Dupré (2012)	52	113	58
Griffiths and Stotz (2013)	60	109	45
Birch and Okasha (2015)	51	21	10
Pradeu (2019)	62	70	36

*Note:* “Biology” denotes a citation in a journal or thesis whose primary readership is biologists or in a general science journal such as PNAS whose readership includes many biologists. “Philosophy” denotes a citation in a journal or thesis whose primary readership is philosophers. “Other” denotes a citation in a journal or thesis whose primary readership is not biologists or philosophers. See SI file for data.

These earlier contributions are mostly ignored by current philosophers of biology, many of whom view POB as originating in the 1960s (see Nicholson and Gawne 2015, Sarkar 2023). This dismissal seems in part to be motivated by the notion that POB can only be done in the style of post-WWII analytic philosophy of science (Reisch 2005, Nicholson and Gawne 2015). Current philosophy of science descends from the logical positivism movement within analytic philosophy (Friedman 1999, Rosenberg 2005). This approach is one in which claims are analyzed as timeless truths and without regard for their social and historical context (Reichenbach 1938, Hanson 1958, Kuhn 1962, Feyerabend 1975, Gadamer 1992). An example is the interpretation of Levins’s (1966) claim about tradeoffs among the attributes of ecological models and his deprecation of computer simulation models in ecology as arising purely from epistemic considerations (e.g., see Orzack and Sober 1993 and Matthewson and Weisberg 2009). Such considerations played a role, but his claims can only be fully understood as arising in part from Levins’s attempt to avoid professional extinction. Computer modeling was being touted in the 1960s as the future of ecology (e.g., Slobodkin 1961, Watt 1962), but Levins then had no access to a computer at his university (Harold Heatwole, University of New England, personal communication, July 2019).

There is some collaborative work in recent POB that reflects direct influence of philosophers on biologists. For example, philosopher Sober contributed important clarification of the variety of hypotheses one can have about the power of natural selection to influence trait evolution to Orzack and Sober’s (1994) work on how to test adaptationism. Other examples of the influence of philosophers include Wilson and Sober’s (1989) work on multilevel selection and Wagner and colleagues (2019) and Love and Wagner’s (2022) work on stress-induced evolutionary innovation.

A broader assessment of whether POB has influenced biology can be gained from citation counts. Consider this sample of well-known articles and books written by prominent philosophers of biology: Hull (1976), Sober (1984, 1988), Dennett (1995), Bechtel (2006), Okasha (2006), Godfrey-Smith (2009), Dupré (2012), Griffiths and Stotz (2013), Birch and Okasha (2015), and Pradeu (2019). As shown in table 1, each has been cited in biology or general science journals, and so there is evidence that POB has had *some* influence on biology. For example, Griffiths and Stotz (2013) have been cited 60 times in biology venues, 109 times in philosophy venues, and 45 times in other venues. We believe that our citation counts likely under-represent the number of citations in philosophy relative to biology (because the Web of Science does not record citations in books, which are more central to research in philosophy than in biology). Either way, the *extent* of influence of POB on biology cannot be answered by this kind of citation analysis.

Some philosophers of biology claim that POB has had an *important* influence on biology. For example, Haber and colleagues (2010) claim that (p. 184) “Over the last few decades, philosophy has had an important impact on biology” and that (p. 185):

philosophers have been instrumental in establishing theoretical biology as a field by collaborating with scientists, publishing in science journals, and taking up conceptual questions at the heart of the biological enterprise.

O’Connor (2020, p. 72) echoes Haber and colleagues’ claims:

Philosophers of science are generally trained to look at science with a critical eye. If this is combined with genuine engagement within an area of science, as in the case of evolutionary game theory, it is unsurprising that the outcome is critique and development of the methods in question. In the best cases, this has led to an improvement of the science at hand. Notably, in the literature surveyed here, this improvement has only been possible because philosophers have gained mastery over the tools of evolutionary game theory. In other words, these are not critiques from without, but critiques from within by sets of academics with interdisciplinary training.

In order to assess whether claims of influence like these are true, one must examine the *potential* domain of citations, that is, we also need to search “in the dark,” which means counting articles, books, and theses that plausibly could contain a citation because of their subject matter but do not. The existence of citations is not by itself evidence of important influence. For example, Orzack and McLoone (2019) defined the potential domain of citations for Levins (1966) as being items cataloged in Google Scholar that had “biology” and “modeling” in their abstract or title. They note that this database (p. 79):

….lists approximately 20,800 articles published in 2017 that contain “biology” and “modeling” in their abstract or title. Of these, 78 cite Levins’ article.

Given this domain of potential citation, Levins’s article appears to have little influence, and we suspect that many works in POB have less influence on biology than Levins’s article. See Pradeu and colleagues (2024, pp. 400–402) for a different but important analysis of the influence of philosophy on biology and other scientific disciplines; they do not consider the potential domain of citation.

Haber and colleagues’ claim that philosophers have been instrumental in “establishing theoretical biology as a field” is incorrect, at least if “theoretical biology” is understood in the way biologists do (e.g., Waddington 1968, Krakauer et al. 2011). For example, in an article addressing “The challenges and scope of theoretical biology,” Krakauer and colleagues do not mention POB and cite only one professional philosopher (Ernest Nagel). We note in regard to O’Connor (2020) that contributions by philosophers of biology to evolutionary game theory are almost all published in journals such as *Philosophy of Science* and in books for philosophers (as indicated by the literature she cites). Most of this literature is likely unknown to the evolutionary biologists who work with game-theoretic models, much less to most evolutionary biologists. There appears to be little contribution by philosophers “within” a scholarly space that is jointly recognized by biologists and by philosophers of biology as including both groups.

A recent claim about Richard Levins by Heger and colleagues (2024) goes beyond a claim that POB has influenced biology. They write (p. 8):

Levins was notable in the sense that he was an ecologist who also seriously engaged with the philosophical literature, so he was able to adopt a philosophical approach to certain ecological problems….

If Levins were “seriously engaged” with the philosophical literature, we imagine this would be evidenced in his writings by citations of articles and books by professional philosophers, especially philosophers of biology. However, only *one* publication (co)authored by Levins contains citations of the philosophical literature in the way that Heger and colleagues appear to define it. In a few publications, Levins cites Hegel, Engels, Marx, and works of Marxist political philosophy such as Cornforth (1963) and Ollman (1993, 2003) or mentions a philosopher such as Descartes without citation. Levins does not appear to be seriously engaged with the philosophical literature. Instead, his philosophy appears to have arisen from his engagement with scientists. For example, Levins (1998, p. 264) writes that he got “excited about dialectics” by reading the articles of scientists such as Haldane and Bernal. The one exception mentioned above is Haila and Levins (1992), which contains citations of work by philosophers such as Diderot, Frege, Kant, and Nagel, among others. However, all of the text sections containing these citations were written by Haila (University of Tampere, personal communication, 7 February 2025).

The above claims indicate that there is a misperception by philosophers of biology of the extent of influence of POB on biology. Why is the influence of POB on biology not greater than it is? One reason is that the disciplinary norm in philosophy about discovering insights that are not dependent on empirical knowledge is at odds with the doing of biology, which is built upon a base of contingent facts, and in which theory is often solely viewed as a tool to guide empirical analyses (e.g., Crick’s 1988, p. 142 statement that “The job of theorists, especially in biology, is to suggest new experiments.”).

Substantive engagement with biology by philosophers of biology is also discouraged by disciplinary constraints. The career prospects of a philosopher of biology almost always lie within a Department of Philosophy, most members of which likely work in areas of philosophy lacking direct engagement with now central scientific practices such as the testing of hypotheses by the application to data of modern statistical methods such as likelihood or Bayesian analysis of generalized linear models. Accordingly, attempts to formulate theories that are useful to biologists are disincentivized in two ways. First, such contributions require effort to acquire the requisite biological knowledge. Second, even successful conceptual contributions to biology may not be considered philosophically interesting, possibly due to their dependence on empirical details, and may be underappreciated due to the lack of biological expertise among philosophical colleagues (Sober 2022). For a philosopher of biology whose professional fate is decided by other philosophers, hewing to the norms of philosophy is less risky than attempting to combine philosophy and biology. This seems unlikely to change, at least without change in universities, such as employing philosophers of biology within Biology Departments; this occurs rarely now.

Despite disciplinary and institutional disincentives, there are current efforts by philosophers of biology to make philosophically informed contributions to biology. An example of these efforts is the “philosophy in science” movement (Laplane et al. 2019, Pradeu et al. 2024). We applaud this important endeavor although at present its constituency is mainly philosophers of biology (e.g., 33 of the 41 speakers at the Fifth PhilInBioMed Network Meeting and Workshop “Philosophical Engagement with Biology and Medicine” held at the University of Bielefeld, 9/30-10/2 2024 were philosophers by training and/or affiliation). In light of the above, we provide the following:

## Suggestions for philosophers who want to contribute to philosophical biology

1. Justify engagement with philosophical biology by its capacity to improve biology. Do not justify engagement with a topic by pointing to its interest to philosophers, or by a generic appeal to interdisciplinarity, or by apparent thematic overlap.
2. Understand that conceptual analysis needs to make a difference to scientific reasoning and practice. The development and clarification of biological concepts is best when based upon actual biology as opposed to imaginary counterfactual scenarios and thought experiments (Hull 1989).
3. Attain at least the level of comprehension of biology possessed by a senior undergraduate major in biology.
4. Publish normative claims about biology in biology journals, not just in philosophy journals.
5. Attend and present work at biology conferences. Collaborate with biologists.
6. Ensure that articles or books about a philosophical issue in biology are reviewed by a biologist with relevant expertise.
7. Do not claim what author X means (without documentation), as in “what Smith really means here is ….” Accept potential ambiguity as a part of human communication.
8. Anchor a descriptive claim about biology in the actual practice of biology (De Regt and Dieks 2005). Engage with current biology and not just biological authorities from the past (e.g., Darwin).
9. Understand that claims by biologists need to be understood in their social and historical context in addition to their epistemic context.
10. Avoid appeals to authority of biologists just because they support POB as an endeavor.

Examples of adherence to some of these include Sober (2004) who engages with the debate over methods used to infer phylogenies (suggestions 1–4 and 8), Birch and Okasha (2015) who engage with the debate over kin selection and provide useful clarification of concepts such as Hamilton’s rule, multilevel selection, and inclusive fitness (suggestions 1–4 and 8), and Gouvêa and Brigandt (2023) who analyze the concept of homology (suggestions 1–4, 8, and 10). All three also likely adhere to suggestion 6.

We now describe some examples of POB that do not exemplify adherence to most or all of these suggestions. As above, we focus on the content and not on the creators.

One example is work advancing the claim that explanations invoking fitness and natural selection are not causal explanations. Instead, they are said to be merely statistical summaries of causes at the level of individual births and deaths (Matthen and Ariew 2002, Walsh et al. 2002) that merely reflect the interests of modelers (Walsh et al. 2017). This claim, known as “statisticalism,” has stimulated extensive debate among philosophers of biology (see Pence 2021). Given that a key concept in evolutionary biology is involved, one might expect that this debate has garnered significant attention from biologists. This does not appear to be the case. Consider, for example, Matthen and Ariew’s (2002) article “Two ways of thinking about fitness and natural selection.” It has been cited 187 times (see table 1). Yet, only 27 citations appear in biology venues, whereas 150 appear in philosophy venues.

One likely reason for this lack of attention is that the debate does not engage with conceptual issues in current evolutionary biology. In addition, verbal toy models are mainly used in the debate (rather than the mathematical models of evolutionary genetics), which limits the relevance of such work for evolutionary biologists (Gildenhuys 2019). The debate over statisticalism is part of a longstanding philosophical debate about whether higher-level descriptions are just summaries of lower-level descriptions and whether reduction could, in principle, be carried out (Pence 2021). The practice is not to offer proposals for how any such reduction is to be carried out, or to reflect on its biological implications. As such, arguments for statisticalism reflect lack of adherence to suggestions to philosophers 2, 4, and 8.

Another example that reflects a failure to adhere to suggestions found in both of our lists concerns recent work on “agency.” This work has been carried out by philosophers of biology (e.g., Walsh 2015, Fábregas-Tejada et al. 2024, Moss 2024, Švorcová 2024) and by biologists (Kauffman 2000, Levin 2022, Newman 2023, Moczek and Sultan 2023).

Agency is said to be “the capacity to make goal-directed changes to one’s self and environment” (Ball 2023, p. 3). Sultan and colleagues (2022) claim that agency is a “definitive property of all living systems” (p. 4), a “system” property that is non-mechanistic and that provides explanations that “cannot be furnished from the mechanism perspective” based on study of the components of the system (p. 8), and a key phenomenon overlooked in current evolutionary biology. Other authors claim that the purported need to go beyond genetic reductionism or neo-Darwinism requires the use of agential explanations (e.g., Nadolski and Moczek 2023, Noble and Noble 2023, Walsh and Sultan 2024, Watson 2024). These claims are problematic (see DiFrisco and Gawne 2025 for discussion).

The work on agency by biologists does not meaningfully engage with the work in philosophy related to causal mechanisms (suggestion to biologists 6), This is reflected in the claim that system-to-component explanations are non-mechanistic. Philosophers have not shown that engagement with agency will improve the doing of biology (suggestion to philosophers 1). Some also appear not to engage with the relevant biology (suggestions to philosophers 3 and 8).

We note that suggestion 2 to philosophers concerning conceptual analysis and the need for it to make a difference in scientific reasoning and practice also applies to the contributions of biologists. An example of adherence to this suggestion is Doolittle’s (1984, pp. 129–133) discussion of how conceptual clarification of the nature of individual and of group selection generates predictions about the evolution of transposable elements. An example of lack of adherence to suggestion 2 is Levins and Lewontin’s advocacy for “dialectical” biology (see above). No matter what one thinks about the merits of this approach, their 1980, 1985, and 2007 works contain little discussion of its practical implications for biological research.

## Should philosophers of biology aim to contribute to philosophical biology?

There is potential for productive deployment of philosophy in biology, but this will not be realized by making room in biological institutions, publications, and conferences for POB *as it is currently practiced*. The practice of philosophers of biology must change if they are to contribute to useful clarification of concepts and development of theories. Equally as important is that biologists accept the essential role of concepts in our understanding of nature and incorporate this understanding into research practice.

The disconnect between philosophy and biology is deep-rooted, and they will not be easily reconnected. We believe the *only* effective way to encourage biologists to engage with conceptual approaches from philosophers is to demonstrate that this engagement leads to better biology.

Of course, one can believe that POB does not need to contribute to biology in order to be worthwhile (cf. Williamson 2024). This is an understandable attitude for areas of philosophy dealing with concepts internal to philosophy, such as truth or morality. It is less compelling when the concepts under investigation are external to philosophy such as fitness or adaptation. What is the value of creating within philosophy a “duplicate conceptual space” containing domesticated versions of concepts used in biology?

We believe that the philosophical practice of forming such duplicate conceptual spaces is tied to the disciplinary agenda to analyze and integrate concepts from multiple areas of inquiry so as to create a “philosophy of” all areas of human endeavor (Kitcher 2023). Many philosophers in the naturalist tradition (e.g., Godfrey-Smith 2014, Rouse 2015) appear to agree with Sellars (1962, p. 35) that

The aim of philosophy, abstractly formulated, is to understand how things in the broadest possible sense of the term hang together in the broadest possible sense of the term.

The notion is that although science may contribute facts to the bigger picture, the understanding of how everything hangs together is supposed to occur within philosophy. This conception of disciplinary identity makes philosophy the ultimate arbiter of what concepts mean and of which viewpoints are justified, even if this arbitration is based on domesticated versions of scientific concepts.

For POB, the value of operating in a duplicate conceptual space has never been justified. It is sometimes said that distance from the biological details helps philosophers to see the big picture. Godfrey-Smith (2014, p. 4) writes, invoking Sellars:

It is foolish for philosophy to place itself above science, but it can certainly step back from science and gain an outsider’s viewpoint. This is necessary, in fact, for philosophy to be able to pursue the task of seeing how everything hangs together.

See also Pigliucci (2013, p. 295). There is great value in “seeing how everything hangs together,” but it is not clear that this goal can be attained by “gaining an outsider’s viewpoint.” Gaining an outsider’s viewpoint risks loss of understanding and relevance. It is entirely possible to be close to the biology *and* to look at the big picture. This is evidenced by the biologists who have made important contributions to the big picture in biology as well as to POB because of their deep knowledge of biology (e.g., Lewontin 1970, Mayr 1988, Ghiselin 1997, Doolittle 2010, Wagner 2014).

It is illustrative to consider what the reception by philosophers would be of work by biologists to provide an outsider’s viewpoint on questions in philosophy such as the nature of truth. We suspect that such efforts would be unwelcome except if the work were done with an insider’s level of engagement with philosophy.

For POB, the defects of operating in a duplicate space of domesticated scientific concepts are clear. Significant effort is devoted to developing toy versions of biological theories that result in little or no improvement of insight as compared to the original theories. Theoretical assertions in the duplicate space become divorced from evidence, which is the basis for confirming and disconfirming assertions. The risk is that workers in that space speak with the authority of science while leaving behind the justificatory basis that comes from empirical connection with nature. Fortunately, there is a better use for the skills acquired by philosophers of biology: they can greatly contribute to the creation of a new philosophical biology. This will require deeper understanding of and engagement with biology.

## Supplementary Material

biag016_Supplemental_File

## Data Availability

The data supporting this study are available as an online supplementary file.
